# Advanced Polymeric Nanoagents for Oral Cancer Theranostics: A Mini Review

**DOI:** 10.3389/fchem.2022.927595

**Published:** 2022-06-14

**Authors:** Guan-Meng Zhang, Shao-Chen Nie, Zhao-Yuan Xu, Ya-Ru Fan, Mai-Ning Jiao, He-Jing Miao, Su-Xia Liang, Ying-Bin Yan

**Affiliations:** ^1^ Department of Oromaxillofacial-Head and Neck Surgery, Tianjin Stomatological Hospital, Hospital of Stomatology, Nankai University, Tianjin, China; ^2^ Tianjin Medical University, Tianjin, China; ^3^ Tianjin Key Laboratory of Oral and Maxillofacial Function Reconstruction, Tianjin Stomatological Hospital, Hospital of Stomatology, Nankai University, Tianjin, China; ^4^ Department of Operative Dentistry and Endodontics, Tianjin Stomatological Hospital, Hospital of Stomatology, Nankai University, Tianjin, China

**Keywords:** oral cancer, nanomedicine, polymer nanoagents, cancer theranostics, targeted delivery

## Abstract

Oral cancer is one of the most common tumours in the world threatening human life and health. The 5-years survival rate of patients with oral cancer has not been improved significantly for many years. The existing clinical diagnostic methods rarely achieve early diagnosis due to deficiencies such as lack of sensitivity. Most of the patients have progressed to the advanced stages when oral cancer is detected. Unfortunately, the traditional treatment methods are usually ineffective at this stage. Therefore, there is an urgent need for more effective and precise techniques for early diagnosis and effective treatment of oral cancer. In recent decades, nanomedicine has been a novel diagnostic and therapeutic platform for various diseases, especially cancer. The synthesis and application of various nanoagents have emerged at the right moment. Among them, polymer nanoagents have unique advantages, such as good stability, high biosafety and high drug loading, showing great potential in the early accurate diagnosis and treatment of tumours. In this review, we focus on the application of advanced polymeric nanoagents in both the diagnosis and treatment of oral cancer. Then, the future therapy strategies and trends for polymeric nanoagents applied to oral cancer are discussed, with the hope that more advanced nanomedical technology will be applied to oral cancer research and promote the development of stomatology.

## Introduction

Cancer is the number one killer that threatens human life and health. Head and neck tumours are the sixth most prevalent cancer type in the world, among which oral cancer is the most common, accounting for 40% (Sung et al., 2021). Despite the advances in oral cancer research over the past few decades, its 5-years survival rate has not significantly improved and still hovers around 50% (Chang et al., 2013). The main reason for the poor prognosis of oral cancer patients is delayed treatment. Only one-third of oral cancer patients are diagnosed at an early stage, with the majority being diagnosed at advanced stages because of the lack of obvious symptoms earlier (Nonaka and Wong, 2018).

The clinical diagnostic methods of oral cancer mainly include biopsy, magnetic resonance imaging (MRI), computed tomography (CT) and positron emission tomography (PET), while biopsy is still the definitive diagnostic method (Keshavarzi et al., 2017; Abati et al., 2020). The treatment of oral cancer depends on its stage. The conventional treatment for early-stage oral cancer is surgical resection, while advanced stage treatment requires a combination of surgery, chemotherapy and/or radiotherapy (Fang et al., 2007; Naruse et al., 2016). However, many traditional chemotherapeutic agents has been limited because of their low bioavailability, inability to specifically identify tumour cells and easy clearance in the blood. Other therapies, such as immunotherapy, gene therapy, photothermal therapy (PTT), photodynamic therapy (PDT), etc., are still under ongoing research.

**FIGURE 1:**
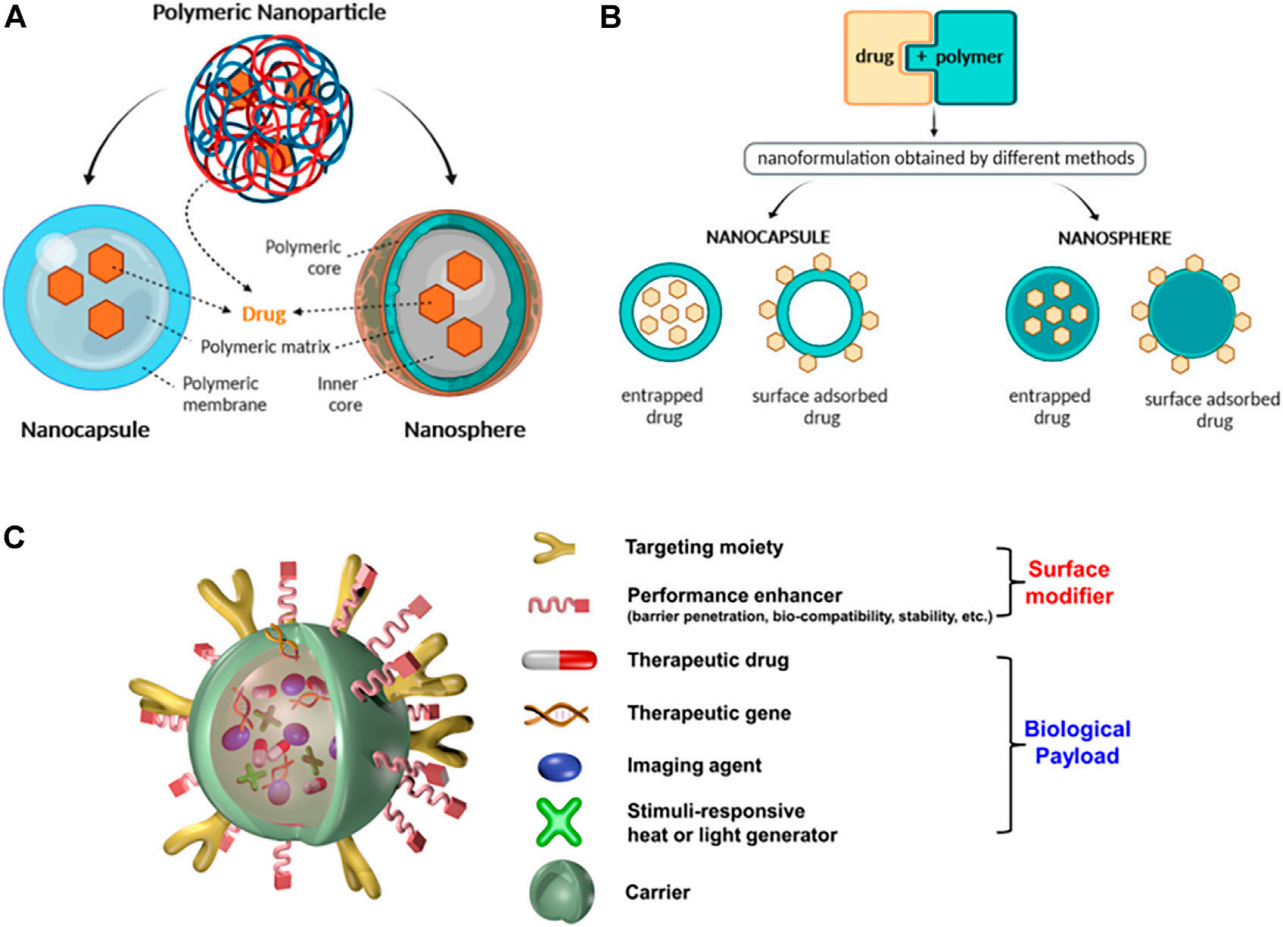
**(A)** Schematic illustration of the structure of nanocapsules and nanospheres. **(B)** Different modes of association of drugs with nanospheres and nanocapsules. [**(A,B)** Reproduced from (Zielińska et al., 2020) with permission from the Multidisciplinary Digital Publishing Institute]. **(C)** Schematic illustration of a multifunctional nanocomposite. Reproduced from (Lim et al., 2015) with permission from American Chemical Society.

Nanomedicine was first proposed by scientists in 2000 (Wagner et al., 2006). The advent of nanomedicine technology has greatly changed the diagnosis and treatment of cancer. Nanomaterials are particles at the nanometre scale that have great potential in the field of medicine due to their special material properties (Lucky et al., 2016; Yu et al., 2020). In the last decade, dozens of drug products containing nanomaterials have been approved by the Food and Drug Administration (FDA) for clinical use (Bobo et al., 2016). Generally speaking, particles between 1 and 100 nm in size in any dimension are called nanoparticles (NPs). NPs usually include liposomes, dendrimers, gold NPs, magnetic NPs, quantum dots, polymeric NPs, etc., Nanomaterials serve as carriers for drug delivery. The unique structure of NPs can be used to deliver fluorescent dyes, chemotherapy drugs, photosensitisers or other biological materials, overcoming the limitations traditional diagnostic and therapeutic processes (Zheng et al., 2021). Due to the leaky vasculatures surrounding fast-growing cancer tissues, NPs carrying anticancer agents can be absorbed by tumour cells through the enhanced permeability and retention (EPR) effects, resulting in local accumulation and cytotoxicity of the tumour cells (Nakamura et al., 2016; Greish, 2010). It can also be conjugated with the corresponding antibodies, peptides, aptamers and small molecules to enhance targeting efficiency and reduce systemic toxicity (Haider et al., 2020). These are the main forms of passive and active targeting of nanoagents. To date, a variety of inorganic and organic/polymer nano-materials for oral cancer research have been reported, including NPs based on metallic and metal oxide materials, quantum dots, solid lipid NPs and polymer-based NPs (Mishra et al., 2018; Su et al., 2019; Soleymani et al., 2020). While each of these well-studied nanoagents has merits, they also have demerits. For instance, the long-term health risks of metal and metal oxide NPs in clinical application remain unknown, and solid lipid NPs are restricted by their poor drug loading capacity (Ruiz-Pulido et al., 2021). Among various kinds of nanoparticles, polymeric nanoparticles have received a lot of attention in tumor research due to their better biosafety and specific drug accumulation effect (Green et al., 2008; Sionkowska, 2011). In the past, non-biodegradable polymers (e.g., polymethyl methacrylate, polyacrylamide and polystyrene) were commonly used to fabricate nanomaterials, but such polymers are difficult to degrade and can lead to chronic inflammation. Nowadays, degradable materials are used as a good alternative to this challenge (Vijayan et al., 2013). Polymeric NPs are prepared from natural polymers (e.g., chitosan and hyaluronic acid) and synthetic polymers [e.g., poly (propylene-co-glycolide) and polyethylene glycol] in a core-shell structure, with hydrophilic blocks forming the shell and hydrophobic blocks forming the core of the nanoparticles (Elsabahy and Wooley, 2013). The size and surface characteristics of nanoparticles are turned by their preparation methods. At present, several preparation methods have been developed and can be divided into two groups, i.e., methods based on the polymerization of monomers and methods using preformed polymers. It is crucial to choose the most suitable preparation method for polymer NPs depending on the specific properties required. After the effective nanoparticles have been synthesised, they are purified by filtration, centrifugation and dialysis techniques (Crucho and Barros, 2017). According to their morphology, polymeric NPs are classified as nanocapsules and nanospheres (Guterres et al., 2007). Unlike other nanocarriers, polymeric nanoparticles can encapsulate the drug within a polymeric oily core (nanocapsules) or disperse the drug in a polymeric matrix (nanospheres) (Crucho and Barros, 2017). Their advantage is that the special core-shell structure allows specific delivery of drugs or fluorescent molecules to the focal area, Figures 1A,B (Zielińska et al., 2020). After the drug released, the polymer matrix is usually degraded to water and non-hazardous molecules containing hydrogen and nitrogen, and is excreted from the body (Parveen and Sahoo, 2008). Their unique properties, such as non-toxicity, water solubility and easy modification, make them promising nanomedicine candidates for a wide range of applications in oncology research, Figure 1C (Lim et al., 2015; Banik et al., 2016; Choudhury et al., 2019).

Although there have been many review articles summarising the development of oncology research, rather limited ones focus on the molecular/NP design and recently developed new mechanisms of the organic/polymer photothermal nanoagents. In this review, we will focus on the research progress based on polymeric nanoagents in the integration of oral cancer diagnosis, treatment and theranostic. We analyse and forecast the current trends and the future treatment strategies, in the hope that more nanotechnology will be applied to oral cancer research and will promote the development of stomatology.

## Polymeric Nanoparticles for Oral Cancer Diagnosis

**FIGURE 2:**
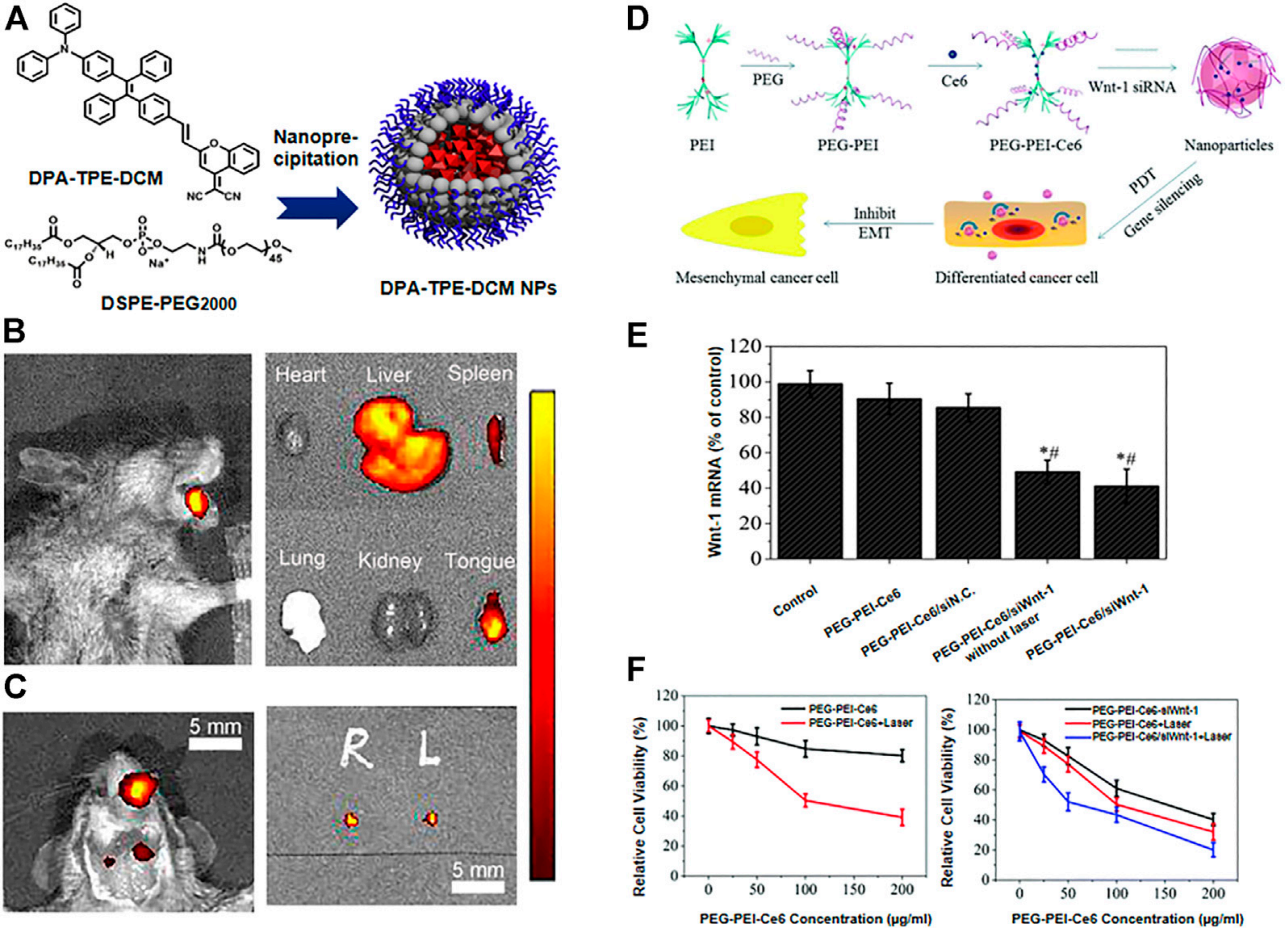
**(A)** Schematic illustration of the preparation of DPA-TPE-DCM NPs. **(B)** Fluorescence imaging of tongue squamous cell carcinoma-bearing mice and *ex vivo* fluorescence images of major organs and tongue after intravenous administration of DPA-TPE-DCM NPs. **(C)** Fluorescence imaging of the mice neck and sentinel lymph nodes helped extract from the mice under the guidance of fluorescence. [**(A–C)** Reproduced from (Zhang et al., 2022) with permission from the Royal Society of Chemistry]. **(D)** Schematic illustration of PEG-PEI-Ce6/siRNA nanoparticles and the mechanism of the effects they exert. **(E)** The expression levels of Wnt-1 mRNA determined by RT qPCR. **(F)** *In vitro* cellular phototoxicity of PEG-PEI-Ce6/siWnt-1 against oral cancer cells. [**(D–F)** Reproduced from (Ma et al., 2017) with permission from the Royal Society of Chemistry].

There is strong evidence that early diagnosis and treatment can lead to a reduced mortality rate in oral cancer (Petersen, 2009). The advantages of polymer fluorescent nanoprobes such as high sensitivity, non-invasiveness and good biocompatibility, make them ideal for imaging (Chan and Wu, 2015). Polymeric nanoagents function as nanocontrast agents or fluorescent probes in the early diagnosis and imaging of oral cancer. For example, Shanavas et al. produced hybrid NPs with a magnetic poly (lactide-co-glycolide) (PLGA) nanoparticle core, where the surface was modified with a folate-chitosan (fol-cht) conjugate shell, and used them as an MRI contrast agent. The hydroxyl (-OH) and amine (-NH_2_) functional groups on the surface of chitosan are extremely reactive, allowing for facile surface modification through complex chemical processes. They showed that the imaging contrast of the targeted group (folic acid receptor) was significantly better than that of the non-targeted group, facilitating the detection of early oral cancer (Shanavas et al., 2017). Inspired by polymeric drug delivery carrier systems, excellent brightness, biodegradability and low toxicity dyes such as Indocyanine green (ICG), Methylene Blue have been conjugated with polymeric nanoagents with special properties that overcome the limitations of poor stability, rapid *in vivo* clearance and low cellular uptake, achieving the early diagnosis through bioimaging of tumours. (Hill et al., 2015). ICG encapsulated in polymer nanoparticles shows significant improvement in both stability and PDT/PTT effect. Poly (styrene-co-maleic anhydride) (PSMA) is an amphiphilic polymer that can be used to encapsulate organic dyes to improve their chemical stability and biocompatibility. Chen et al. designed a kind of nanoparticles in which they encapsulated ICG with PSMA to form ICG@PSMA by self-assembly method. *In vitro* and *in vivo* studies have shown that ICG@PSMA NPs have strong NIR fluorescence, good biocompatibility, low toxicity and excellent photothermal properties. And they found that ICG@PSMA NPs have great potential in different types of cancer (Chen et al., 2021). However, achieving high brightness with dye-loaded polymer NPs requires loading large quantities of fluorescent dyes, which can cause the occurrence of aggregation-caused quenching (ACQ) and limit the brightness of dye-loaded polymer NPs. With the development of nanomaterials, the aggregation-induced emission (AIE) effect was discovered (Zhang et al., 2021). Zhang et al. synthesised an AIE material named phenylene and tetraenzenewere-dicyanomethylene-benzopyran (DPA-TPE-DCM) and applied it to the optical diagnosis of early oral cancer. The probe showed good biocompatibility and shows a high signal-to-noise ratio when applied *in vivo*. Under the guidance of fluorescence, the orthotopic tongue carcinoma in mice was successfully detected, as well as the mapping of sentinel lymph nodes smaller than 2 mm, Figures 2A–C (Zhang et al., 2022). Bioimaging reveals the biological processes involved in early carcinogenesis, helps detect small tumours at an early stage and aids in the assessment of resection margins during surgery. Nanotechnology provides the means for more accurate imaging of lesions and has greatly advanced the field of oncology.

## Polymeric Nanoparticles for Oral Cancer Prevention and Therapy

The occurrence of oral cancer is a relatively complex process, involving multiple genetic and cellular alterations (Chi et al., 2015; Li et al., 2020). In oral cancer, chemical prophylaxis is considered effective in reversing, preventing or inhibiting the malignant transformation of precancerous cells. Natural plant compounds, such as flavonoids and astragalus, are of great interest due to their rich biological activity and medicinal value, making them promising chemopreventive agents (Iriti and Varoni, 2013; Glenny et al., 2010). However, the low bioavailability and poor solubility of such plant compounds lead to limited clinical application (Singh et al., 2014). To overcome this problem, the use of polymeric nanoparticle drug delivery systems for packaging biologically active plant compounds for oral cancer prevention has been clinically explored (Gohulkumar et al., 2014). Licorice is a perennial plant with well-known pharmacological properties and is considered as an adjunct to the drug treatment of many local and systemic diseases. Glycyrrhetinic acid (GA) is a kind of active compounds extracted from licorice and has significant anti-cancer properties (Roohbakhsh et al., 2016). Cacciotti et al. examined the cytotoxicity of GA on human oral squamous cancer cells (OSCC) and normal human gingival fibroblasts (HGFs) with two delivery systems: chitosan (CS) and poly (lactic-co-glycolic) acid (PLGA) based nanoparticles and polylactic acid fibers (FBs). They used a one-step osmosis based methodology to prepare CS-PLGA-based NPs (GA-NPs) loaded with GA, which were non-toxic to HGFs and had a low median toxic concentration of 200 μmol/L against human OSCCs, both of which were superior to GA-FBs (Cacciotti et al., 2018). When oral cancer progresses to an advanced stage, clinical treatments like radiotherapy and chemotherapy are mostly applied. Since most chemotherapy drugs are easily cleared by the reticuloendothelial system (RES) in blood circulation and are lowly water-soluble, poorly biocompatible, and lowly targeted, they often fail to cure tumours and instead cause serious side effects, such as vomiting, fever, allergies, and hair loss. Polymer nanoagents have been used as drug transport carriers to improve the stability of drugs, control targeted drug delivery, make the concentration of drugs in the lesion site constant and uniform. El-Hamid et al. showed that pegylated liposomal doxorubicin (Doxil) had a higher apoptotic effect on CAL-27 cells than free doxorubicin with fewer side effects (El-Hamid et al., 2019). In another study, Gupta et al. synthesised PLGA NPs encapsulating the model radiosensitising drug docetaxel, presenting higher toxicity to human oral cancer cells than free docetaxel (Gupta et al., 2018). Polymer nanoagents are inherently biocompatible and biodegradable, and have an extended residence time at the local site. Encapsulating the active drug into polymer NPs can overcome the problems of poor drug solubility and low bioavailability, enhance drug stability, thereby increasing efficacy and reducing side effects.

Optical therapy, such as PDT and PTT, is an emerging method of tumour treatment (Ou et al., 2019). PDT has been officially approved by the FDA for the treatment of localised oesophageal cancer (Lee and Baron, 2011). The photosensitiser (PS) is activated by light in the presence of oxygen, leading to the generation of reactive oxygen species (ROS). Some researchers have used PS coupled with polymeric NPs for photodynamic therapy of oral cancer. Wang et al. designed an effective ROS-sensitive delivery carrier for chemical photodynamic therapy, named polyethylene glycol-polycarbonate-thioketal doxorubicin (PEG-PBC-TKDOX). Doxorubicin (dox) was covalently modified to self-destructive polymeric micelles for drug release *via* light-triggered ROS. The polymer system improved the efficiency of chemical photodynamic therapy and reduced off-target toxicity (Wang M. et al., 2019). In addition, nanoparticles combined with laser can be effectively used in photothermal therapy, attacking tumour cells without significant damage to other cells. In a study by Ren et al., synthesized organic compound (C3) was equipped with indocyanine green (ICG) into polyethylene glycol-polycaprolactone (PEG-PCL) to form hybrid nanoparticles (PEG-PCL-C3-ICG NPs) for combined PTT and PDT treatment of tumours under 808 nm laser irradiation. *In vitro* and *in vivo* experiments showed that PEG-PCL-C3-ICG NPs were significantly more effective than PTT or PDT separately, and had better performance and lower cytotoxicity compared to conventional PTT agents such as Au nanorods (Ren et al., 2017). Based on the special properties of nanoplatforms, some researchers have combined chemotherapy and optical therapy as a multimodal treatment approach, achieving good results in the treatment of oral cancer. Wang et al. developed a novel drug delivery system, called human serum albumin indocyanine green-cisplatin nanoparticles (HSA-ICG-DDP NPs), to ensure site-specific drug delivery/release and reduce the systemic toxicity of chemotherapy, demonstrating the synergistic effects of PDT, PTT, and chemotherapy with *in vitro* and *in vivo* experiments. As for the *in vivo* treatment, HSA-ICG-DDP NPs accumulated in tumour tissues and exhibited stronger antitumour effects compared to ICG, HSA-ICG and DDP treatments, significantly improving the therapeutic efficacy (Wang et al., 2019a). Combination therapy is a current trend in oncology treatment that improves the results and reduce side effects. Multifunctional polymeric nanocarriers are ideal materials for combination therapy.

Gene therapy has been studied in oncology treatment for several years, but the results achieved have not been significant. The advent of nanomedicine has greatly facilitated the development of gene therapy. In photodynamic therapy, activation of epithelial-to-mesenchymal transition (EMT) can lead to tumour recurrence and progression. Some investigators have enhanced the effect of PDT by inhibiting the Wnt/β-catenin signaling pathway involved in EMT progression using nanocarriers carrying corresponding small interfering RNA (siRNA). Ma et al. efficiently delivered Wnt-1 siRNA into the cytoplasm of PDT-treated oral cancer cells using polyethylene glycol-polyethyleneimine-chlorin e6 (PEG-PEI-Ce6) NPs. PEG-PEI-Ce6 nanoparticle-mediated PDT in combination with Wnt-1 siRNA significantly inhibited cell growth and enhanced the killing effect on cancer cells, Figures 2D–F (Ma et al., 2017). The application of nanomaterials has overcome the limitations of the traditional treatments and has provided new options for the treatment of oral cancer patients.

## Polymeric Nanoparticles for Oral Cancer Theranostics

In traditional clinical practice, the time-phased medical model of diagnosis followed by treatment is cumbersome. Nanopolymer drug delivery platforms have been used to integrate the process of diagnosis and treatment as a new direction of tumour theranostics (Lim et al., 2015). In some investigations, highly sensitive fluorescence for diagnosis and multimodal therapy have been integrated into a single system through nanodrug delivery platforms to achieve diagnosis and treatment of oral cancer. Wang et al. designed and synthesised a multimodal near infrared (NIR)-II nanoprobe, [4,4'-((6,7-bis(4-(hexyloxy)phenyl)-[1,2,5]thiadiazolo [3,4-g]quinoxaline-4,9-diyl)bis (thiophene-5,2-diyl))bis (N,N-diphenylaniline)] TQTPA loading cis-dichlorodiammine platinum (CDDP) (HT@CDDP) by hyaluronic acid. They proved to have good stability and water solubility and exhibited biocompatibility and low systemic toxicity. *In vitro* and *in vivo* experiments demonstrated that the NPs have good imaging capabilities and are capable of drawing the outlines of orthotopic tongue tumors and metastatic lymph nodes as small as 1 mm in nude mice by IR-808 under NIR exposure. Also, the NPs can be used as a multimodal therapeutic agent combining photothermal therapy with chemotherapy to achieve combined chemotherapy-photothermal treatment (Wang et al., 2019b). The strategy of treatment with simultaneous visual diagnosis simultaneous facilitates real-time monitoring of the treatment’s effects and the formulation of individualised treatment plans. Thus, it is considered a promising strategy for early clinical cancer diagnosis and treatment that deserves further investigation.

## Conclusion

Herein, we reviewed the progress of research on polymeric NPs in oral cancer prevention, diagnosis and treatment. Polymeric NPs provide new platforms and ideas for the diagnosis and treatment of oral cancer that are worth exploring in greater depth. During the last decade, research in nanotechnology in the field of medical oncology has been in full swing. However, the polymeric NPs also have drawbacks: research indicates that some polymeric NPs are prone to hazardous degradation and toxic monomer aggregation, necessitating further research into their synthesis and chemical characteristics. Importantly, there are still some pressing issues in the study of polymeric nanoagents for oral cancer applications. Changes in the tumour microenvironment (e.g., temperature and pH) often affect the effectiveness of nanoplatform-based drug delivery systems. In this context, NPs regarding the tumor microenvironmental response are being studied extensively, still not in oral cancer. The large discrepancies between the results of *in vivo* and *in vitro* experiments have raised major doubts concerning the effectiveness of nanosystems in humans. In addition, the targeting efficiency of polymer NPs *in vivo* has not achieved the desired effects. Due to the lack of specific markers in oral cancer, since some proteins that are overexpressed on the surface of tumour cells also exist in normal cells, the manner to further improve the efficiency of passive and active targeting remains to be elucidated.

There is no denying that nanotechnology, especially polymeric nanocarrier platforms, has the potential to be the most effective and beneficial form of treatment and diagnosis of cancer in the future. Further research is needed to translate nanotechnology concepts into practical applications. In the coming years, it will play a key role in early tumour detection, diagnosis and treatment procedures. However, polymer NPs-based diagnosis and treatment of oral cancer still has a long future.
